# Usability of Graphical Visualizations on a Tool-Mounted Interface for Spine Surgery

**DOI:** 10.3390/jimaging7080159

**Published:** 2021-08-21

**Authors:** Laura Schütz, Caroline Brendle, Javier Esteban, Sandro M. Krieg, Ulrich Eck, Nassir Navab

**Affiliations:** 1 Chair for Computer Aided Medical Procedures, Department of Informatics, Technical University of Munich, Boltzmannstrasse 3, 85748 Garching b. München, Germany; javier.esteban@tum.de (J.E.); ulrich.eck@tum.de (U.E.); nassir.navab@tum.de (N.N.); 2 Clinic and Polyclinic for Neurosurgery at Klinikum Rechts der Isar, TUM School of Medicine, Technical University of Munich, Ismaninger Strasse 22, 81675 München, Germany; sandro.krieg@tum.de

**Keywords:** tool-mounted interface, surgical navigation, spine surgery, pedicle screw placement, medical augmented reality, visualization, usability

## Abstract

Screw placement in the correct angular trajectory is one of the most intricate tasks during spinal fusion surgery. Due to the crucial role of pedicle screw placement for the outcome of the operation, spinal navigation has been introduced into the clinical routine. Despite its positive effects on the precision and safety of the surgical procedure, local separation of the navigation information and the surgical site, combined with intricate visualizations, limit the benefits of the navigation systems. Instead of a tech-driven design, a focus on usability is required in new research approaches to enable advanced and effective visualizations. This work presents a new tool-mounted interface (TMI) for pedicle screw placement. By fixing a TMI onto the surgical instrument, physical de-coupling of the anatomical target and navigation information is resolved. A total of 18 surgeons participated in a usability study comparing the TMI to the state-of-the-art visualization on an external screen. With the usage of the TMI, significant improvements in system usability (Kruskal–Wallis test *p* < 0.05) were achieved. A significant reduction in mental demand and overall cognitive load, measured using a NASA-TLX (*p* < 0.05), were observed. Moreover, a general improvement in performance was shown by means of the surgical task time (one-way ANOVA *p* < 0.001).

## 1. Introduction

An aging society and a growing share of sedentary work have led to a rapid rise in the prevalence of degenerative spinal disorders. Exemplary indications causing spine instability, which, in turn, can lead to bone deformation or nerve damage are disc degeneration, spondylolisthesis or scoliosis [1]. Among different surgical approaches, spinal fusion is identified as a cost-effective and good-quality treatment strategy for spine degenerative diseases and is also being used for a wider range of indications [2], making it one of the most frequently performed procedures worldwide [3].

In this type of surgery, an artificial reconstruction of the spine’s stability is performed by introducing a transpedicular screw–rod system into the spine [4]. The precise placement of the pedicle screws is one of the most intricate tasks because deviations from the targeted trajectory can result in injuries of the spinal cord, nerve roots or blood vessels [5].

So-called navigation systems are used for enhanced localization and precise placement of surgical instruments to maximize the precision of pedicle screw insertion [6]. An improved outcome and reduced radiation exposure are prevailing reasons to use these systems [7]. In recent years, robotic systems for spine surgery, such as ExcelsiusGPS [8] and Mazor X [9], have become commercially available. These solutions are likely to improve the precision and predictability of patient treatment even more.

**Figure 1:**
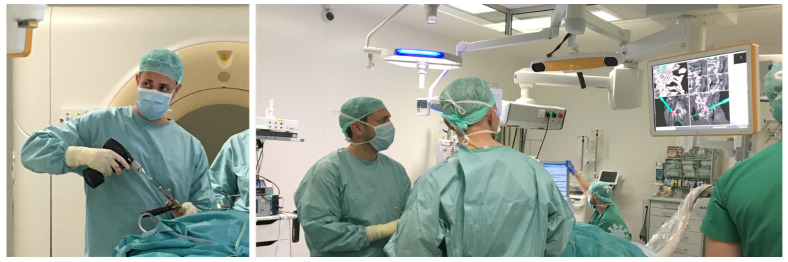
Physical de-coupling of navigation information and situs. Neurosurgeons using a traditional navigation system for pedicle screw placement during spinal fusion surgery.

However, the complexity of use and the disruption of the surgical workflow are predominant factors for the conservative percentage of navigated spine surgeries [10]. According to Härtl et al., only 11 percent of spine surgeons use navigation systems on a regular basis [10]. When using navigation, the operators must split their attention between the navigation information, displayed on an external screen, and the surgical site (situs) [11], adding to the complexity of use (Figure 1).

Shifting the surgeons’ attention from an external screen back to the patient is a widely-researched topic in the scientific community. The application of augmented (AR) and mixed reality (MR) in the medical context aroused interest several decades ago [12].

Half-mirror displays and projectors have been used to superimpose pre-operative images onto the patient for anatomic navigation.

Navab et al. developed a surgical AR technology enabling video-augmented X-ray images by extending a mobile C-arm with a video camera called CAMC [13]. In their work, as in our study, instrument axis alignment was evaluated. However, AR images are displayed using a mirror attached to the C-arm and no external navigation is used, while we propose localization of the visualization unit on the surgical instrument. Habert et al. [14] provided a new variation of the original CAMC, using an attached RGBD camera to the X-ray detector. These concepts were later used by Philips Medical to offer an advanced visualization solution for spine surgery [15]. Similar to the CAMC solutions, the Philips system provides monitor-based visualization, requiring the surgeon to move their attention from the surgical scene onto the external monitor.

Other works that have focused on approaches using projectors are from Gavaghan et al. [16,17], who presented a portable projection system for the projection of navigational information onto the patient’s skin. This system assists in targeting incision points for percutaneous needle placement and also displays navigation guidance onto anatomical structures, such as the surface of a liver phantom.

Besides half-mirrors and projectors, technological advancements have enabled optical see-through head-mounted displays (OST-HMD); several surgical navigation systems with head-mounted tracking and displays have been proposed [18,19,20].

Liebmann et al. evaluate the precision of a novel navigation method that is characterized by the additional in-situ visualization. The focus of this work is the investigation of the accuracy of a lumbar pedicle screw insertion combined with the visualization information provided by the Microsoft HoloLens in contrast to state-of-the-art navigation systems [21]. The study examined 3D augmented views using a Microsoft HoloLense, whereas we propose visualizations on a 2D display.

As an alternative to the work mentioned above, some developments focus on fixing displays or mobile devices onto the surgical instrument itself.

The potential of adding a smartphone as a man–machine interaction device is shown by Gael et al. [22]. Several combinations of displays and anatomical information are explored in different experiments of needle placement. Benefits, such as local exploration around the current tool position or an easier and safer access to the target, are reported.

The efficacy of a novel navigation system with an iPod-display for total knee arthroplasty (TKA) is presented and discussed by Mullaji et al. [23]. Additional anatomical information, such as the resection level or slope, is presented on the iPod screen that is held by hand. Despite a negligible expenditure of time, compared to conventional TKA, the precision of this navigation solution is reported to be comparable to conventional navigation systems. In contrast to both studies, our work presents visually reduced information independent of an external monitor as also explored by the following works.

**Figure 2:**
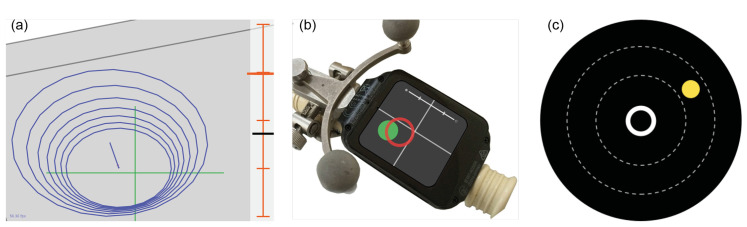
Comparison of graphical tool-mounted guidance interfaces: (**a**) Kassil et al. (**b**) Herrlich et al. (**c**) Our approach.

Kassil et al. investigated an approach of a tool-mounted guidance display. The performed study proves that the tool-mounted guidance display yields more accurate tool placement than the conventional interface on an external monitor [24]. They compared two tool-mounted interfaces: one uses a graphical navigation interface with a target line and concentric rings, and the other, in addition to the graphical elements, augments the image of a camera installed on the tool as a background. (Figure 2) Their results showed that the camera view had no significant improvement for the application. Our approach builds on this work, as we also use graphical interfaces for orthopedic tool alignment. However, we present a visualization where the target point is always fixed in the center of the screen, and no target line nor depth scale is shown. Our work is an attempt to reduce the complexity of navigational interfaces even further. We hope to show that such improvements allow the surgeon to better focus on the execution of the guided action and, therefore, increase their accuracy, while improving the usability.

Additional work that introduced an approach for measuring cognitive load, user preference, and general usability is presented by Herrlich et al. [11]. A navigation approach for needle placement that mounts a smartwatch display directly onto the instrument is presented. During an empirical study with non-clinical experts, the benefits of the system in terms of reduced cognitive load and improved general usability while achieving the same performance as the external monitor were reported. They proposed a 2D guidance that maps the shaft and the tip of the needle onto two colored circles on the smartwatch display. Other than this work, our interface only contains one moving object: the tool’s position in respect to the centered target trajectory.

Unlike Kassil et al. and Herrlich et al., we conducted an extensive user study with medical experts from the field to evaluate both performance, as targeted by Kassil et al., and usability, as focused by Herrlich et al. To the best of our knowledge, the performance, cognitive load and usability have not been evaluated for a graphically-reduced tool-mounted interface for the task of drill-guide alignment with surgeons.

Against this background, the main objective of this work is to present a navigation approach comprising intra-operative digitization and intuitive graphical guidance for a computer-assisted screw placement procedure. Maximizing the amount of navigated spinal procedures, thus minimizing complication rates for patients, presents prevailing factors for the clinical motivation of this work.

In this paper, we propose a user-centered navigation interface for spinal surgery. The approach consists of a tool-mounted interface (TMI), attached to a shaft for surgical tool guidance. This way, the TMI enables the display of navigation information in the surgeon’s field of view.

A proof-of-concept study to show the usability advances of this user-centered navigation system is the primary focus. The concept of the TMI could be comprehensively evaluated by two experiments for the task of angle alignment. Two different visualizations for angle orientation of the surgical tool were tested with 18 physicians against the visualizations used in the clinical routine. In contrast to the early findings published by Brendle et al. [25], the usability advances of the proposed concept are evaluated in depth and discussed in detail. In addition, an updated angle visualization and a preliminary usability study for a proposed depth control visualization during drilling holes into vertebrae were added to this article.

## 2. Materials and Methods

**Figure 3:**
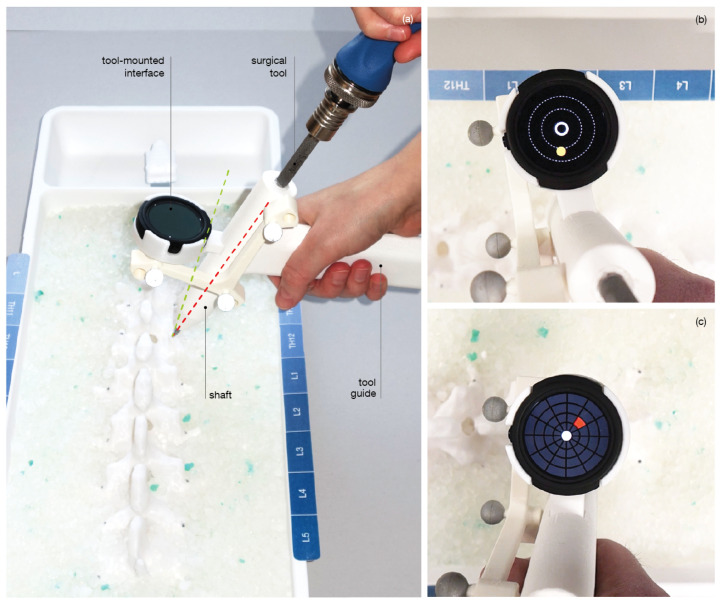
(**a**) Tool-mounted interface, green line: target trajectory, red line: current tool trajectory. (**b**) Close view of Circle Display. (**c**) Close view of Grid Display.

To prevent the physical separation of navigation information and the surgical site, we propose the use of a tool-mounted interface (TMI). The concept of the TMI is an outcome of user-centered design [26]. The navigation concept comprises a handle, a shaft for tool guidance, a tracking array and a mount for the visualization unit (Figure 3). The custom-made handler is designed to be used in combination with a surgical instrument, allowing a visualization unit, such as the TMI to be mounted on it. This idea introduces a shift in perspective that is distinct from the solutions used in the routine clinical practice. This approach keeps the user’s attention on the surgical site rather than directing it away from the patient to an external monitor. At the same time, it does not force the user to wear a head-mounted display and, therefore, is easily integrated into the surgical environment and workflow.

### 2.1. Hardware and Software Setup

The system is made up of a workstation, a Polaris Vicra (NDI, Ontario, Canada), the TMI and a Ticwatch E (Mobvoi, Beijing, China). The Ticwatch is clipped to the visualization unit mount to display the instrument-integrated visualizations. A Polaris Vicra infrared tracking system tracks the arrays affixed to both the phantom and the instrument. The calibration between the marker and the tool is known by construction and is verified using a pivot calibration. The software utilizes a client-server architecture running between the Ticwatch E and the workstation. The server is executed as a plugin on the ImFusion Suite (ImFusion GmbH, Munich, Germany (https://www.imfusion.de)), which processes the tracking data and transmits them to the Ticwatch to generate the visualization. The frame rate is 50 Hz on the workstation and 40 Hz on the Ticwatch visualizations.

With the intention of enhancing the usability of spinal navigation user interfaces (UI), a reduction in cognitive load is targeted. To be compliant with this requirement, a graphically reduced approach to interface design is chosen. In contrast to traditional navigation UI, a view at the situs and the visualization unit is provided. This view favors the employment of a subjective perspective and not an objective view of the instrument trajectory in relation to the patient anatomy on an external screen (as is used in traditional navigation). Providing a subjective view of the instrument position in relation to the ideal trajectory on an integrated display is pursued by the TMI.

The visualization under evaluation is designed for the task of instrument angle alignment. This sub-task of the pedicle screw placement procedure is chosen, as it defines the angle at which the hole is drilled into the vertebra and directly influences the accuracy of the pedicle screw position. During angle alignment, the instrument tip is already positioned on the bony anatomy of the vertebra. Thus, the instrument’s movement is limited to rotations within three dimensions inside the space of a hemisphere. A top view of the hemisphere allows for an overview of the trajectory, without losing sense of the current localization. Thus, this perspective was chosen and abstracted from 3D to 2D during the UI design. Since a top view of a hemisphere is a circle, the display shape was chosen accordingly to match the UI.

**Figure 4:**
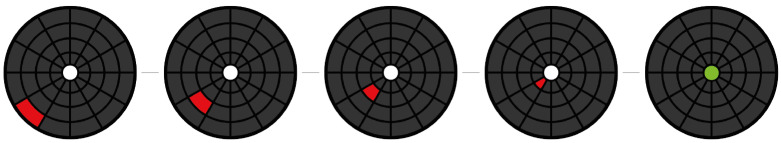
User Interface Design of Grid Display.

The proposed visualization strategy aims at reducing the complexity of the navigation information during pedicle screw placement for the operating surgeon. Two visually reduced interfaces displaying abstract 2D guidance were designed (Figure 4). The two visualizations Circle Display and Grid Display essentially map the real-time 3D Cartesian orientation of the TMI to a polar coordinate system centered at the pre-planned insertion trajectory. In such a manner, the relative angular distance between the planned and the actual trajectory is intuitively displayed on the tool-mounted 2D screen.

**Circle Display** The Circle Display UI comprises a circle element that dynamically moves across the underlying background in accordance with the live pose of the TMI, which is computed as outlined in the preceding section. By orienting the TMI such that the circle-shaped element enters the center ring of the UI, the user attains the desired trajectory. The correct positioning is indicated to the user by providing complementary visual feedback: the circular element changes from a yellow to a blue color, and an additional highlighting element is superimposed around the central ring.

**Grid Display** The second UI design is characterized by a grid structure that divides the surface of the display into 12 segments of a circle. In addition, four concentric rings of varying radii are arranged in a regular pattern. Depending on the relative position of the TMI with respect to the planned path, one of the respective grid patches is colored red. (Figure 4) By moving the TMI in the denoted direction of the red-flagged field, the system guides the user in the direction of the desired angular orientation. As soon as the user has reached the pre-planned target alignment, the middle circle is illuminated in green.

**Traditional External** The navigation interface used in state-of-the-art spinal navigation systems displays three slices with normal directions $tool_{X,Y,Z}$ and application point $tool_{tip}$. The Traditional External UI used in our study emulated the state-of-the-art visualization. It was generated using the ImFusion Suite. The pre-planned pedicle screw insertion path is superimposed on all three planes (axial, coronal, sagittal) of the patient CT scan as a red line. The TMI’s position is represented using a green line. The pre-planned, optimal orientation is attained when both the red and the green line intersect on all three individual planes.

### 2.2. Validation Setup and Experiments

The primary focus of this usability study is the evaluation of the TMI for the task of tool angle alignment within the pedicle screw placement procedure. To compare the proposed approach with the traditional visualization approach, two sets of controlled experiments were conducted. The study was executed in a closed environment inside the university hospital Klinikum rechts der Isar, Munich, Germany (https://www.mri.tum.de/) to evaluate the potential benefits of the proposed approach. A controlled scenario for task execution was generated. This controlled setup allowed for the collection of reliable data on the user’s performance in regard to the task time, Euclidean distance, overall task load, system usability and UI design. The aim of the study is the yielding of information on the following two research questions:

- Does the proposed user-centered navigation concept enhance the usability and performance of the medical task for the surgeon, compared to the state-of-the-art navigation? (Experiment 1)
- Is the execution of the alignment task easier with a tool-mounted interface or with an external screen? (Experiment 2)

Experiment 1 was focused on the examination of the medical procedure and the yielding of clinical results. The participant group consisted of eighteen experienced surgeons. It investigated the performance and usability of the TMI, compared to the state-of-the-art navigation on a realistic spine phantom.

Experiment 2 was employed to evaluate the two main distinctions of the proposed over the traditional approach in an isolated manner—the two main aspects being the in situ visualization offered by the TMI on one hand, and the developed UI on the other hand. To evaluate whether the location of the proposed visualizations affects the system performance and usability, the user was presented with an alignment task using the same pair of visualizations (Circle and Grid), both on the TMI and the external screen. The distribution of the participants in both experiments followed a block randomization. The following hypotheses were framed. Each assumption is compared to the respective visualization presented on the external screen.

- Compared to the state-of-the-art navigation system: (Experiment 1)
  **H1.** Participants using the TMI for angle alignment experience reduced cognitive load and improved usability.
  **H2.** Participants using the TMI for angle alignment achieve the planned trajectory faster and with a shorter Euclidean path.

- Compared to the same visualizations on an external screen: (Experiment 2)
  **H3.** Participants using the TMI for angle alignment experience reduced cognitive load and improved usability.
  **H4.** Participants using the TMI for angle alignment achieve the planned trajectory faster and with a shorter Euclidean path

A complementary experiment was conducted to gather preliminary results of the TMI for the task of drilling. In addition to the evaluation of the angle alignment task in Experiments 1 and 2, this small experiment with non-significant results looked at the usability and design of one proposed depth control visualization. The complementary experiment was supposed to give insights into the potential of the TMI for sub tasks outside the angle alignment task. The choice to additionally investigate depth feedback was based on the surgical workflow wherein the surgical tool is first aligned, and once correct alignment is achieved, the drilling process begins, thus presenting a logical subsequent guidance visualization to investigate.

#### 2.2.1. Experiment 1

**Figure 5:**
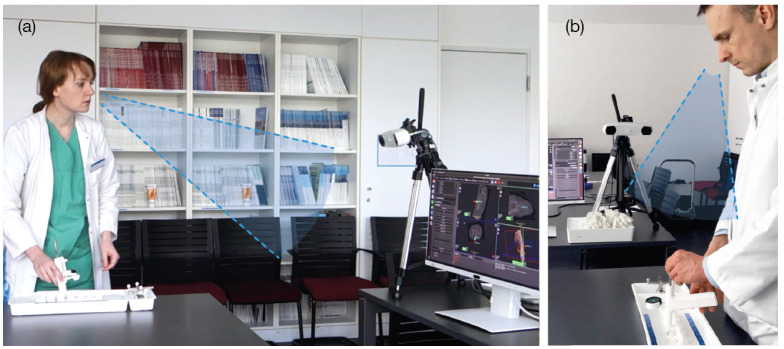
(**a**) Execution of Experiment 1, neurosurgeon using the external screen. (**b**) Senior neurosurgeon using the visualization on the TMI.

**Participants.** A total of 18 surgeons (8 females and 10 males) volunteered in the study (Figure 5). All of the practicing doctors were experienced in using traditional techniques for the visualization of 3D data (i.e., CT, MRI). Their mean age was 30 ± 3.7 std. The participants could be grouped into two levels of experience:

- Attending physicians (senior surgeons) from the Department of Neurosurgery at Klinikum rechts der Isar: skilled users regarding pedicle screw placement and spinal navigation systems.
- Residents (junior surgeons) and medical student interns from the Department of Neurosurgery at Klinikum rechts der Isar: familiar with spine anatomy and the surgical procedure, but with little to no experience in executing pedicle screw placement and operating spinal navigation systems themselves.

None of the participants had previous experience using the proposed navigation system. However, 14 participants indicated to be familiar with using surgical navigation systems, and 15 participants reported to have executed pedicle screw placement before. At the end of two weeks of experiments, 4 attending physicians, 11 residents, and 2 medical student interns participated in the experiment.

**Figure 6:**
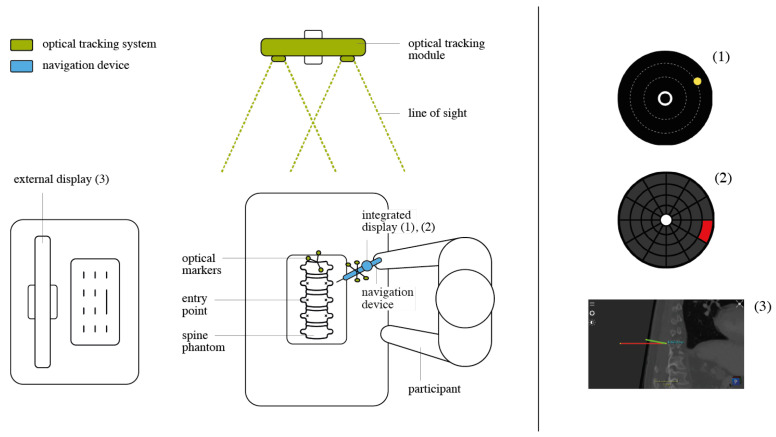
Setup of Experiment 1. (**1**) Circle Display, (**2**) Grid Display, (**3**) transversal view of Traditional External.

**Experiment Setup** A moderated usability study was employed. The experiment was executed as a physical session including a moderator, a software operator and one participant at a time. The moderator instructed the participant on the task performed with the product under evaluation, accompanied by a questionnaire referring to the task and the product. Experiment 1 evaluated the traditional approach to navigation presented on an external display versus the proposed TMI approach. A total of three visualizations (Circle Display, Grid Display, Traditional External) were evaluated on a model of the lower lumbar spine (levels Th11 to L5) (Figure 6).

The model was 3D printed from a CT scan of a human spine and calibrated to the optical tracking system. To guarantee anatomical conformity, realistic pedicle screw insertion paths were defined by a senior neurosurgeon on the CT of the printed spine phantom prior to the study execution. The trajectories represented the anatomically correct path of insertion for each right and left pedicle screw at the levels Th11 to L5 of the spine and amounted to 14 distinct trajectories that were equally spread on both sides of the spine. The entry point of each screw trajectory was physically marked on the phantom.

In addition, scales were added on both sides of the spine model to indicate the levels and sides. During the experiment, these scales helped the participants to find the entry points that were communicated verbally, e.g., “Next trajectory Th12 left side”. First, the user was instructed to place the tip of the TMI onto one of the predefined entry points on the spine phantom. Then, the physician was asked to align it in the right anatomical, pre-planned trajectory, using one of the visualization techniques. This alignment was repeated four times on two randomized trajectories for each side of the spine. The same task was repeated for all three visualizations (Circle Display and Grid Display on the TMI, Traditional External on the external screen). To avoid data distortion, the order of visualizations was randomized.

#### 2.2.2. Experiment 2

**Figure 7:**
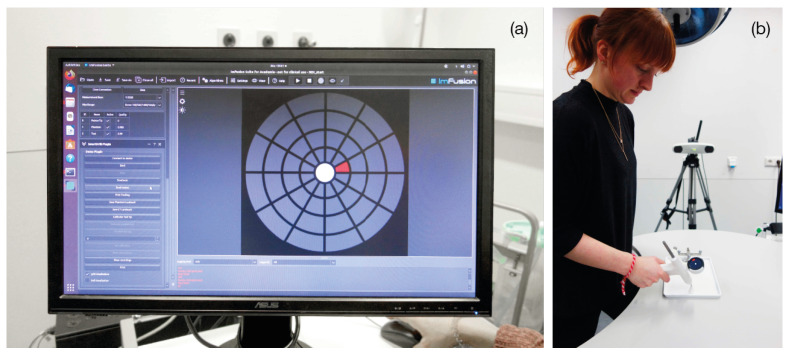
(**a**) Execution of Experiment 2, participant using Grid External. (**b**) Participant using Grid Display on the TMI.

**Participants** A total of 42 volunteers (15 females and 27 males) participated in the second experiment at a research laboratory at Klinikum rechts der Isar (Figure 7). The mean age was 26.8 ± 3.3. None of them had clinical experience, while 31 stated to having experience using augmented or virtual reality. However, none of the participants had any previous experience using the TMI system or the visualizations under evaluation.

**Figure 8:**
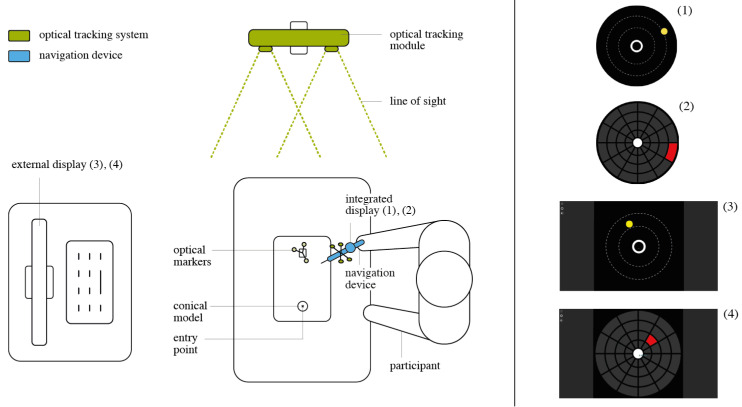
Setup of Experiment 2. (**1**) Circle Display, (**2**) Grid Display, (**3**) Circle External, (**4**) Grid External.

**Experiment Setup** We performed a second experiment to isolate the two main aspects of our solution: the in situ localization of the visualizations and the visualizations themselves. As a combination of the two UI designs (Circle and Grid), and the two displaying locations (integrated and external) four distinct setups were evaluated (Figure 8).

An abstracted use-case scenario was built. A pyramidal 3D model with a single entry point was employed instead of the spine phantom. As another part of the task abstraction, random realistic trajectories were created with a fixed entry point on top of the conical model, contrasting the anatomically right insertion paths in Experiment 1. The participant was asked to position the tip of the TMI onto the given entry point on top of the physical 3D model. Then, the user was asked to align the TMI in the pre-planned trajectory, using the visualization. This alignment task was repeated five times per visualization for each of the four mentioned setup combinations. The order in which the participant used the four visualizations was randomized. The experiment was accompanied by a questionnaire referring to the task and the product.

#### 2.2.3. Complementary Experiment

**Figure 9:**
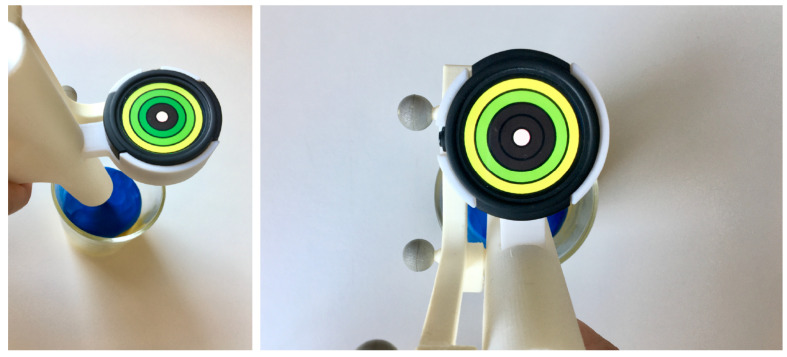
Depth control visualization on the TMI used to insert the handler into modeling clay (blue) to simulate the procedure step of drilling.

**Participants and Experiment Setup.** A moderated usability study was employed to investigate a proposed visualization for tool depth control on the TMI (Figure 9). The guidance was used during drilling into the vertebra to receive live feedback on the depth of the tool with respect to the planned hole depth. A total of 9 male participants with a mean age of 28 ± 3.5 were asked to position the tip of the TMI into a glass containing modeling clay and to insert the TMI into the mass according to the guidance on the Ticwatch screen. The clay was used to simulate the consistency and resistance of bone marrow during the procedure. The depth control visualization showed green rings that lit up one by one the further the tool was inserted into the clay until the innermost circle element turned from white to green, indicating that the ideal depth of the hole had been reached. The task was repeated three times. An accompanying questionnaire referring to the task’s usability, overall task load and the visualization’s design was filled out right after the experiment.

#### 2.2.4. Experimental Variables

Based on the posed hypotheses, the experimental variables were defined. These variables determined the type of measurements performed during the experiment. Their values were examined using statistical analysis, and they enabled the hypotheses validation.

**Euclidean Path and Time** During Experiments 1 and 2, time-stamped poses of the TMI handler were recorded. Time measurement was one of the chosen performance metrics used during the simulation trials. Measuring the task completion time provides information about the efficiency of a product. In the case of navigation usage, the faster the task is completed, the better the experience is for the user. On average, the surgeon executes the task between 4 and 12 times during one procedure and usually has two surgeries a day, resulting in a highly repetitive task, where efficiency becomes predominant according to Albert and Tullis [27]. The task time is measured for each trajectory individually. The time measurement starts when the navigation visualization appears on the screen and ends after the instrument is perfectly aligned for a duration of 2 s. The start and end times are accompanied by the moderator’s verbal cues, “start” and “success”. The time-stamped poses of the TMI also allowed the collection of the Euclidean path traveled by the TMI during task execution. Analyzing the total Euclidean distance traveled provides information on how well the visualizations’ logic was understood by revealing how directly or indirectly the tool was guided toward the final position.

**Cognitive Load and Usability** Additionally, the participants of all three experiments were asked to fill out a questionnaire comprising a NASA-TLX, a System Usability Scale (SUS) and three Design-related Likert scales for measuring the cognitive load, overall usability and perceived user experience (UX) and user interface design. The NASA-TLX (Task Load Index) is a self-reported workload assessment of the executed task. This standard method from the field of human factors engineering provides quantitative data on the cognitive load, allowing an objective comparison between visualizations. Cognitive load refers to the overall task load calculated, using the NASA-TLX questionnaire, for six defined variables (mental demand, physical demand, temporal demand, performance, effort, and frustration) [28]. Usability was calculated using the System Usability Scale (SUS) questionnaire [29]. SUS is a simple, ten item questionnaire that records a user’s subjective assessment of usability. For each item, a five-point Likert scale collects data on the perceived usability of the product. The scales are numbered from 1 (anchored with “Strongly disagree”) to 5 (anchored with “Strongly agree”). SUS yields a single value representing a composite measure of the overall usability of the system under evaluation, yielding results in a range of 0 to 100 in 2.5 increments, wherein 0 represents a very bad usability, and 100 a very good usability [30].

**Design** In addition to the hypothesis-related measures, the design aspects were evaluated. The questionnaire included further questions gathering subjective assessments of the interfaces’ design. The aspects of visual appeal, intuitiveness and interaction were evaluated, using three five-point Likert scales, numbered analogously to the ones used in the SUS questionnaire. In addition, open questions were employed, collecting qualitative data on the visualizations’ design, such as positive and negative aspects during usage.

## 3. Results

### 3.1. Experiment 1

**Figure 10:**
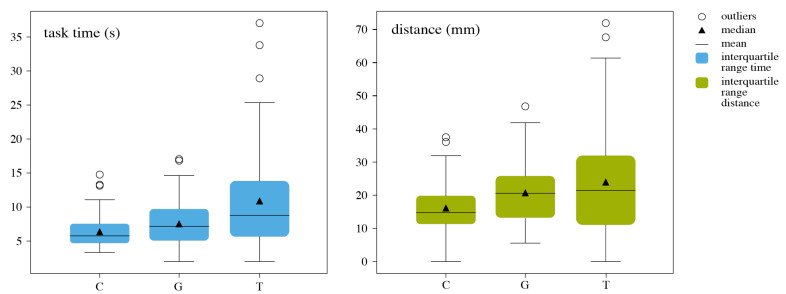
Boxplots of task time and euclidean distance for Circle Display (C), Grid Display (G) and Traditional External (T) for Experiment 1.

**Euclidean Path and Time** The entire Euclidean path of the tool was analyzed. For this purpose, 3D time-stamped poses of the tool’s shaft were recorded during the experiments. The Euclidean path was computed as the total sum of the Euclidean distances between each successive pair of poses. This value expresses the cumulative travel distance of the tool’s shaft until the alignment is successful. Intuitively, this measure provides insights into how directly the user moved the tool toward the final pose. (Figure 10) (chi-squared distributed (*p* < 0.001)). The time values showed a normal distribution (*p* < 0.05). Outliers with values outside of mean ± 2 ∗ std were excluded from the data set. A one-way ANOVA test for time and the Kruskal–Wallis test for distance were employed to measure significance.

The proposed approach achieved significantly better results with respect to time (*p* < 0.001), compared to the traditional approach. Circle Display performed better, compared to the traditional method, for both distance (*p* = 0.0107) and time (*p* < 0.001).

**Experience Level** The time data were grouped by the participants’ experience level, indicated by the doctors in the questionnaire accompanying Experiment 1. Experience was defined as the absolute number of times the participant used a spinal navigation system. The surgeons had four experience categories to choose from:

1. Never–10 times.
2. 11–50 times.
3. 51–250 times.
4. More than 250 times.

The means and standard deviations of the time values grouped by experience level can be found in Table 1. The analysis using a one-way ANOVA on the time values between pairs of visualizations revealed that participants with no to little experience in using spinal navigation performed the tool alignment task significantly faster when using the proposed approach. For this group, the task time when using Circle Display and Grid Display was significantly faster (p<0.05) than when using Traditional External. All other groups with higher experience levels and prior training in using spinal navigation systems showed no significant (p>0.05) difference in task time between visualizations. It can be concluded that the proposed visualizations are more intuitive, helping the least experienced group of surgeons to adapt to and execute the task faster than when using the traditional navigation interface.

**Spine Anatomy** It was shown by a recent study that the accuracy and reliability of CT-based spinal navigation varies between the three areas of the spinal column: cervical (C), thoracic (Th) and lumbar (L). The rate of screw placement accuracy within these areas was 93% for cervical, 96.33% for thoracic, and 96.4% for the lumbar spine as reported in a study by Kumar et al. [31]. An analysis of the times and Euclidean distances by vertebrae (Th11 to L5) was performed to look for a similar phenomenon. However, when grouping the data into the thoracic and lumbar regions, no significant differences (p>0.05) between areas were found.

**Cognitive Load and Usability** The recorded NASA-TLX and SUS data showed a chi-square distribution for all different variables (p<0.001). For each of the NASA-TLX variables and the SUS score, a Kruskal–Wallis test was performed pairwise between the visualizations. The analysis revealed that the use of both Circle Display and Grid Display reduced the mental demand (p<0.05) and cognitive load (p<0.05) significantly, compared to Traditional External. The usability (SUS score) for Circle Display (79.4 ± 14.1 std) was significantly better (p<0.05), compared to Traditional External (68.7 ± 14.7 std) (Table 2). When comparing the two proposed designs Circle Display and Grid Display against each other, no significant difference (p>0.05) was reported.

**Design** A chi-square test was run for all three design variables. A *p*-value lower than our expected significance level was shown (*p* < 0.001), indicating a statistically significant association between the three visualizations and respective design variables. The results of the variable visual appeal (Likert scale [1, 5] higher is better) revealed that Circle Display (3.8 ± 1.1 std) was perceived as the most visually appealing visualization, followed by Grid Display (3.7 ± 1.3 std) and Traditional External (3.1 ± 0.9 std) (Table 3). A pairwise one-way ANOVA showed no significant difference between the visualizations (*p* > 0.17). The same ranking resulted for the intuitiveness of the visualizations. Only Circle Display was rated as being significantly more intuitive (*p* < 0.01) than Traditional External. Regarding the user experience, the interaction with Circle Display was rated best, Traditional External second best and Grid Display the worst. Again, one-way ANOVA tests for pairwise comparisons were employed. They showed that Circle Display was rated as being significantly more intuitive (*p* < 0.01) than Traditional External. The other tests revealed no significant differences between the pairs. Regarding the answers to the question, “Which visualization would you prefer to use in the future?”, Circle Display and Grid Display shared first place with both being 38.2%. Only 23.5% stated to prefer the Traditional External visualization.

### 3.2. Experiment 2

**Figure 11:**
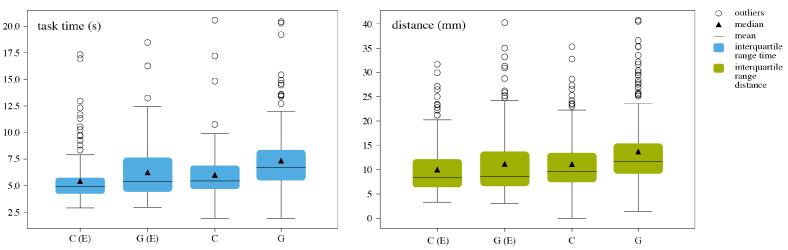
Boxplots of task time and Euclidean distance for Circle External (C(E)), Grid External (G(E)), Circle Display (C) and Grid Display (G) for Experiment 2.

**Euclidean Path and Time** To allow comparison of the different visualizations, the Euclidean distances and total time of each task were categorized based on the visualization used (Circle Display, Grid Display, Circle External, Grid External), as shown in Figure 11. Any outliers with values outside mean ± 2 ∗ std were removed from the sample. Within the four groups (for both time and distance), the normality was tested using D’Agostino’s K-squared test (*p* < 0.001). The sample had a ratio of 2.004 between larger and smaller variances. A single factor ANOVA was used to compare the results in terms of the Euclidean distance and time. Circle External performed best, both regarding time and distance, followed by Circle Display and Grid Display (Table 4). The data revealed that the visualizations on the external screen performed significantly better than the visualizations on the device (*p* < 0.001).

**Cognitive Load and Usability** The NASA-TLX and SUS results showed a chi-squared distribution of the different variables (*p* < 0.001). A Kruskal–Wallis test was used to compare the external visualization group (Circle External and Grid External) with the instrument-integrated visualization group (Circle Display and Grid Display) for all variables of the NASA-TLX and SUS score (Table 4). No significant difference was found in the analysis between the two groups (*p* > 0.05), neither for cognitive load nor for usability; hence, no comparisons between pairs were conducted.

**Figure 12:**
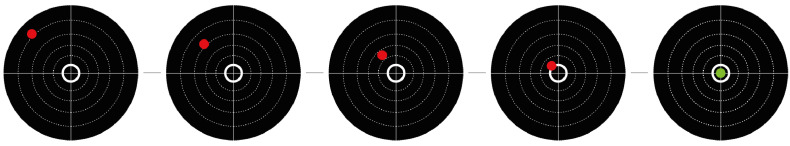
Further development of the Circle visualization based on participant feedback.

**Design**. The analysis of the design scales showed a chi-square distribution for all three variables (*p* < 0.001). Additionally, a single factor ANOVA was run for the pair of newly proposed visualizations. It revealed that Circle was perceived as being significantly more visually appealing (*p* < 0.001) than Grid (Table 5). The same result in favor of the Circle design emerged for the intuitiveness of the visualizations (*p* < 0.001). In terms of user experience during the interaction with the interface, Circle once again proved to be significantly superior (*p* < 0.001) to Grid. Overall, the Circle visualization emerged as the preferred option in all design aspects in Experiment 2. When asked in the open questions section what they liked and disliked about the two visualizations, the participants suggested that the discrete space grid of the Grid visualization felt jumpy at times and that it did not represent the smooth movement of the tool. They positively mentioned the direct translation of the TMI’s movement to the movement of the virtual circle element implemented in the Circle visualization, stating that this continuous movement allows for precise location awareness as well as high sensitivity and control. When asked about what to change to improve the Circle visualization, the participants mentioned adding supporting lines, reducing the size of the moving circle element to better fit into the center ring, changing the color scheme to red and green, and realizing higher movement sensitivity the closer to the target one is. As a result of this user feedback, the Circle visualization was updated as shown in Figure 12.

### 3.3. Complementary Experiment

Due to the small participant group, the experiment’s results are not significant. They provide a rough estimation of whether the concept of the TMI evaluated in detail for angle alignment is also a feasible option for depth control when targeting improved usability.

**Cognitive load and usability.** The depth visualization showed a mean cognitive load (NASA-TLX) of 24 ± 9.7 std, mental demand (NASA-TLX) of 24 ± 18.6 std and usability (SUS) of 75 ± 6.8 std (Table 6). These values are in the same range as the results reported for the task load and usability for Circle and Grid.

**Design.** The design of the depth control visualization for the three variables visual appeal, intuitiveness and interaction was rated as follows: visual appeal, 4.3 ± 0.5 std; intuitiveness, 4.6 ± 0.5 std; interaction, 4 ± 0.7 std. These values are again similar to the ones reported for Circle and Grid. When asked what the participants would improve about the visualization, they mentioned adding a warning signal once the tool overreaches the intended depth and exponential scaling to allow a higher sensitivity of the guidance closer to the target depth.

## 4. Discussion

A tool-mounted interface (TMI) for spinal fusion surgery was presented. Preliminary tests demonstrated significant improvements in favor of the proposed approach in terms of cognitive load, mental demand, and usability (H1). Enhanced user ergonomics resulted in a significant reduction in task time (H2) as well as an increase in performance, measured as the total Euclidean distance and time for the group of novice surgeons. Experiment 2 revealed no significant findings for H3, while H4 was proven to be false, as the results on the external display were significantly better. Among the possible factors influencing this result are the latency added to the visualizations on the TMI, the lower resolution, and the comparatively small size of the smart watch display. This is an interesting result, which contrasts the results of Experiment 1 that show a reduction in cognitive load, improved usability, and superiority in comparison to the traditional approach. This could guide one to improve the resolution and the performance of the attached display. Nevertheless, the principal idea to reduce the number of external displays and to introduce in situ guidance to minimize the user’s cognitive load is still valid.

An additional usability experiment introducing a depth control visualization on the TMI has shown that the TMI solution does not only promise potential for the task of angle alignment, but also for depth feedback during surgical tool insertion.

The grouping of time data by surgeon experience level in Experiment 1 showed that the proposed approach is understood faster and is thus more intuitive. It can be assumed that traditional visualizations have to be learned to achieve the same performance as the proposed approach. This identified benefit makes a strong case for the application of graphical visualizations in surgeon training. The reduced cognitive load of the TMI can help beginners focus on the perception of haptic feedback during instrumentation usage, thereby training their fundamental surgical skills while allowing them to still achieve precise pedicle screw placement by employing surgical navigation.

Despite the known variance in the level of difficulty for pedicle screw placement, depending on the level of the vertebrae, the grouping of time and Euclidean distance by vertebrae for Experiment 1 has not shown significance. Even though the study setup was designed to replicate the real-use scenario, the spine phantom was not comparable to a human body in regard to spine mobility. The high mobility between vertebrae has a major influence on the difficulty of screw placement in clinical practice. The results are not surprising when considering this distinction between the study setup and real clinical application.

When looking at the introduction of the TMI in real clinical application, questions on calibration and system performance may be raised. We propose checking the system’s accuracy intra-operatively with a simple pivot calibration before each operation. Such verification procedures are a common part of the routine workflow of existing navigated solutions, and are done prior to the procedure, usually taking a few seconds. The performance of the tracking system depends on the system used; in our case, we used a common Polaris Vicra. Such systems are already employed in clinical routine, and they offer sub-millimeter precision if used under optimal conditions.

Despite some questions that the initial testing has left open, the TMI has proved its high potential. The decision to focus the presented study on a graphically reduced approach to surgical navigation visualization on a tool-mounted interface allowed for an exploration of possibilities within medical user interface (UI) design and was rewarded with enthusiastic responses from both engineers and clinicians. The presented work has proven that minimalist interfaces, when positioned intelligently to integrate well into the surgical workflow, can achieve the same precision as traditional interfaces for tool alignment tasks while improving the overall usability.

This work opens a path for the research community to further regard user-centered approaches to surgical interfaces and to hopefully contribute to usability advancements in surgical visualizations.

## Figures and Tables

**Table 1:** Surgeon experience level: means and stds for task time (in sec) grouped by experience level (number of times participant has used spinal navigation system) for Circle Display (C), Grid Display (G) and Traditional External (T) for Experiment 1.

Experience Level	Never–10 Times	11–50 Times	51–250 Times	More than 250 Times
C	8.2 ± 2.1	4.6 ± 2.2	9.6 ± 0.6	6.9 ± 1.1
G	7.2 ± 3.5	8.3 ± 3.1	9.3 ± 2.0	6.2 ± 2.7
T	16.0 ± 10.6	7.3 ± 0.2	10.7 ± 4.0	9.3 ± 0.9

**Table 2:** Means and stds for time (in sec), distance (in mm), cognitive load, mental demand (NASA-TLX [0, 100]; lower is better), and usability (SUS [0, 100]; higher is better) for Circle Display (C), Grid Display (G) and Traditional External (T) for Experiment 1.

Exp. 1	Time	Distance	Cognitive Load	Mental Demand	Usability
C	6.4 ± 2.4	16.2 ± 7.5	23.7 ± 15.4	23.6 ± 20.1	79.4 ± 14.1
G	7.5 ± 3.2	20.8 ± 9.9	25.0 ± 14.4	25.8 ± 19.0	76.7 ± 18.1
T	10.9 ± 7.3	24.0 ± 16.7	35.3 ± 12.7	42.8 ± 18.3	68.7 ± 14.7

**Table 3:** Means and stds for visual appeal, intuitiveness and interaction (Likert scales [1, 5]; higher is better) for Circle Display (C), Grid Display (G) and Traditional External (T) for Experiment 1.

Exp. 1	Visual Appeal	Intuitiveness	Interaction
C	3.8 ± 1.1	4.6 ± 0.5	4.2 ± 0.9
G	3.7 ± 1.3	4.2 ± 0.5	3.6 ± 0.9
T	3.1 ± 0.9	3.9 ± 0.7	3.7 ± 0.9

**Table 4:** Means and stds for time (in sec), distance (in mm), cognitive load and mental demand (NASA-TLX [0, 100]; lower is better), and usability (SUS [0,100]; higher is better) for Experiment 2.

Exp. 2	Time	Distance	Cognitive Load	Mental Demand	Usability
Circle Display	6.0 ± 2.2	11.2 ± 5.8	32.2 ± 17.3	33.7 ± 25.3	80.8 ± 13.1
Grid Display	7.4 ± 2.9	13.7 ± 7.1	"	"	"
Circle External	5.4 ± 2.2	10.0 ± 5.4	33.3 ± 17.1	36.9 ± 26.9	80.2 ± 18.1
Grid External	6.3 ± 2.5	11.2 ± 6.8	"	"	"

**Table 5:** Means and stds for visual appeal, intuitiveness and interaction (Likert scales [1, 5]; higher is better) for Experiment 2.

Exp. 2	Visual Appeal	Intuitiveness	Interaction
Circle	4.6 ± 0.6	4.8 ± 0.4	4.6 ± 0.8
Grid	3.6 ± 1.1	4.0 ± 1.2	3.3 ± 1.3

**Table 6:** Means and stds for cognitive load (Cog. Load), mental demand (Mental D.) (NASA-TLX [0, 100]; lower is better), usability (SUS [0,100]; higher is better), visual appeal, intuitiveness and interaction (Likert scales [1, 5]; higher is better) for the depth control visualization.

	Cog. Load	Mental D.	SUS	Visual Appeal	Intuitiveness	Interaction
Depth	24 ± 9.7	24 ± 18.6	75 ± 6.8	4.3 ± 0.5	4.6 ± 0.5	4 ± 0.7

## Data Availability

The data presented in this study are available on request from the corresponding author. The data are not publicly available.

## Abbreviations

The following abbreviations are used in this manuscript:

TMI	Tool-Mounted Interface
AR	Augmented Reality
MR	Mixed Reality
RGBD	Red-Green-Blue-Depth
NDI	Network Device Interface
CT	Computer Tomography
MRI	Magnetic Resonance Imaging
NASA-TLX	NASA Task Load Index
SUS	System Usability Scale
UI	User Interface
UX	User Experience
Th	Thoracic Spine
L	Lumbar Spine
